# Now What? Maintaining Momentum after Achieving Designation as an Age-Friendly University

**DOI:** 10.1093/geroni/igab046.2800

**Published:** 2021-12-17

**Authors:** Diane Martin, Nicole Brandt, Denise Orwig, Barbara Resnick, Daniel Mansour, Elizabeth Galik, John Schumacher, Meredith Upton

**Affiliations:** 1 Geriatrics & Gerontology Education and Research Program, University of Maryland, Baltimore, Baltimore, Maryland, United States; 2 University of Maryland School of Pharmacy, University of Maryland School of Pharmacy, Maryland, United States; 3 University of Maryland, Baltimore, Baltimore, Maryland, United States; 4 University of Maryland, Baltimore, Maryland, United States; 5 University of Maryand School of Pharmacy, Baltimore, Maryland, United States; 6 University of Maryland School of Nursing, Baltimore, Maryland, United States; 7 University of Maryland, Batlimore County, Baltimore, Maryland, United States

## Abstract

The Age-Friendly University (AFU) designation in higher education recognizes the institution’s commitment to a culture of age-inclusivity across programs and policies. While AFU institutions are embracing the demographic shifts in higher education and society at-large, effectively responding to the needs and desires of an increasingly older population requires ongoing acceptance and support from campus leaders to maintain momentum and stay relevant within a dynamic field. This session will describe the intentional and systematic approach utilized by an AFU steering committee to build interest in and develop meaningful collaborations in multiple domains across campus, including at the level of the president. Our initiatives focus on five themes that align with the 10 age-friendly university principles: support for workforce development, broadening community engagement, expanding engagement in aging research and dissemination, addressing barriers related to aging and our physical environment, and facilitating age-friendly efforts across our state. We will present our experiences in expanding awareness of and support for the AFU movement on our campus and share a model for institutions seeking ideas for sustainability of their own initiatives.

